# Emotional labor among team members: do employees follow emotional display norms for teams, not for customers?

**DOI:** 10.3389/fpsyg.2023.1265581

**Published:** 2023-11-30

**Authors:** DaEun Hong, MinSoo Kim

**Affiliations:** School of Business, Hanyang University, Seoul, Republic of Korea

**Keywords:** emotion labor, emotional regulation, team emotional display norms, self-regulatory focus, moderated multi-level polynomial regression, selfless OCB

## Abstract

Emotional labor is typically conceptualized as a process in which individuals regulate their emotions in response to display rules. Most research on emotional labor has focused on the influence of display rules at individual-level perceptions but is rarely examined at the team level. We examine the influence of the shared display rules in teams as emotional display norms. This study considers emotional dissonance as the difference between the positive emotional display norm at the team level and positive emotion at the individual level. To examine the purpose of this study, data were collected from leader-follower pairs within teams and based on a three-wave design. Thus, this study conducted a multi-level polynomial regression analysis and used the response surface methodology to interpret the incongruence effect. The results show that the incongruence effect of emotional dissonance is positively related to surface acting. In addition, the moderating effect of regulatory focus significantly strengthens the positive relationship between emotional dissonance and emotion regulation strategies. The results also show that surface acting strategy is negatively related to selfless Organizational citizenship behaviors (OCB). These findings highlight that emotional display norms play an important role as the standard for emotional experience in teams, and especially with the moderating effect of self-regulatory focus, emotion regulation strategies affect the selfless OCB rating of observers.

## Introduction

Many previous studies on emotional labor have focused primarily on the interaction between employees and customers (Grandey, 2000; Diefendorff et al., 2019). However, in the context of the team achieving common goals, team members experience emotional labor through continuous social interactions (Lam et al., 2021; Bindl et al., 2022). Given that team members’ emotions affect not only their own emotional experiences but also relationships among other team members and team performance, it is important to explore the emotion regulation process of team members (Troth et al., 2012, 2018). This study examines emotional labor based on the interactions among team members.

As an antecedent of emotional labor, the display rule has been considered a formal rule for how emotions should be managed during interactions with customers (Rafaeli and Sutton, 1990; Ashforth and Humphrey, 1993). Individuals use emotion regulation strategies to manage their emotions following display rules. According to Becker and Cropanzano (2015), team members experience emotions that are appropriate to the situation and share emotional expectations as informal rules. Thus, the display rule can be the standard for emotional experience not only when members interact with customers but also when team members interact with each other. Research from this view discussed that the emotional display rule can be shaped informally by being shared within a team but rarely examined the influence of shared emotional display norms within a team.

Studies on emotional labor in teams need to consider not only the emotion regulation experience of team members but also the interpretation of emotions. According to Grandey (2003), a member’s emotions through emotion regulation strategies can be interpreted differently even when expressing the same positive emotion. This is because, even with the same positive emotion, observers perceive the differences in the authenticity of emotion, as positive emotions represent the desire to maintain relationships with others and the pleasantness of interactions (Grandey, 2000; Grandey et al., 2012; Parke and Seo, 2017). Accordingly, positive emotions are not always to be interpreted in the same way (Manokara et al., 2023). The observer interprets subsequent behavior based on the expressor’s positive emotion through an inference process (Van Kleef, 2009). Therefore, it is necessary to consider the effect from the observer’s perspective, especially how a team member’s positive emotion is interpreted differently by the observer, which can affect the evaluation of future behavior.

**FIGURE 1:**
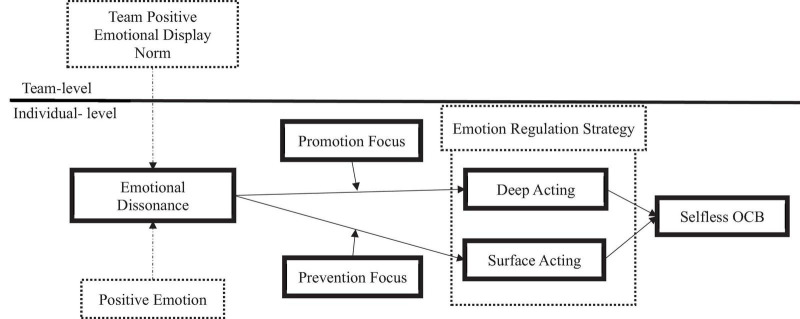
Hypothesized model.

Specifically, this study examines the emotional dissonance of team members as the incongruence between positive emotional display norms at the team level and positive emotions at the individual level. As shown in Figure 1, we verify our hypotheses through a multi-level polynomial analysis, which uses the difference value between the team’s positive emotional display norm and the individual positive emotion in this regard. First, we hypothesize the relationship with emotion regulation strategies when there is incongruence between the team’s positive emotional display norm and the members’ positive emotions. Second, we explore the moderating effect of the regulatory focus on the relationship between emotional dissonance and emotion regulation strategies using a three-dimensional graph based on the response surface methodology. Third, our research examines whether the observer rates the members’ positive emotions differently according to the regulation strategy. This research investigates whether the observer’s interpretation is based on the previous reaction even when the members engage in selfless OCB in the future. Through these, we explore the influence of emotional dissonance according to the shared positive emotional display norm and the antecedent factors and outcomes of emotion regulation strategies from a multilevel perspective. In this respect, we aim to contribute to the literature on emotional labor by shedding light on the emotional regulation process of team members in the service organization and other various organizations.

## Theory and hypothesis

### Team’s positive emotional display norms

Previous research has primarily focused on job requirements regarding emotional display rules (Thoits, 2004; Grandey et al., 2013), particularly formal rules applicable to employees in the service industry who interact with customers (Hochschild, 1983; Ashforth and Humphrey, 1993). Formal display rules include the organization’s expectations, whereas informal display rules include shared display beliefs among individuals (Ekman and Friesen, 1975; Diefendorff and Greguras, 2009; Diefendorff et al., 2011). However, recent research on emotional labor among team members has begun to view emotional display rules as a concept of both formal and informal social norms (Diefendorff et al., 2006; Grandey and Melloy, 2017; Zapf et al., 2021).

Display rules are defined as the standards for individual members’ appropriate emotional experience (Rafaeli and Sutton, 1987; Diefendorff and Richard, 2003). Team members do not only experience emotions but also express and share them in their work teams (Menges and Kilduff, 2015; Gabriel et al., 2020). These team members experience appropriate emotions based on their situations while performing interdependent tasks, following each other’s emotional expectations (Kozlowski and Klein, 2000; Grandey et al., 2012). According to Diefendorff et al. (2011), display rules can be formed by top-down factors, such as the leader’s expectations and work environment. However, they can also be shaped by bottom-up factors, such as through the interaction between team members. Therefore, display rules may become shared attributes among team members at the team level (Becker and Cropanzano, 2015; Menges and Kilduff, 2015). Parke and Seo (2017) suggested that members who constantly interact to attain a common goal experience improved interpersonal relationships, especially through positive emotion, and believe that frequent expressions of positive emotion can increase the pleasantness of the interaction. As such, the team develops display rules for the most appropriate positive emotion in the team context, which may vary among teams (Diefendorff et al., 2011). In addition, shared display norms within the team indirectly affect the display rules perceived by individual members and form a social context that forces them to follow (Feldman, 1984; Rafaeli and Sutton, 1990; Barsade and Gibson, 1998). Therefore, we propose that the team’s positive emotional display norms are shaped as informal rules shared among all members based on formal rules (Kelly and Barsade, 2001; Grandey et al., 2012).

### The effects of emotional dissonance on emotion regulation strategies

Emotional dissonance can be defined as the discrepancy between a team’s positive emotional display norms and an individual’s positive emotions (Brotheridge and Grandey, 2002). In following the team’s positive emotional display norms, members constantly compare whether their positive emotions are appropriate to the group display norms (Diefendorff and Gosserand, 2003). In this comparison process, individuals can recognize the difference between their positive emotions and required positive display norms. According to Festinger (1957), cognitive dissonance is a psychologically unstable state that induces individuals to reduce their dissonance because it causes stress. In other words, individuals who have experienced emotional dissonance attempt to reduce the difference between their positive emotions and the team’s positive emotional display norms (Pugh et al., 2011). However, because display norms are rules for managing team members’ emotions who are pressured to follow them, individual members can try to change their emotions (Ashforth and Humphrey, 1993; Becker and Cropanzano, 2015). Therefore, team members experience emotional labor that regulates their emotions based on shared team display norms through constant comparison processes (Morris and Feldman, 1996; Gosserand and Diefendorff, 2005).

Emotion regulation strategies refer to strategic behaviors in which individuals intentionally manage their emotional experiences and expressions (Gibson and Schroeder, 2002; Diefendorff and Gosserand, 2003). Grandey (2003) described emotion regulation strategies as deep acting and surface acting. Deep acting refers to expressing an individual’s emotions by consciously modifying them to the required emotions, such as display rules, and surface acting refers to hiding one’s emotions and faking them as required (Hochschild, 1983; Brotheridge and Grandey, 2002; Gosserand and Diefendorff, 2005). Thus, Grandey (2000) suggests that display rules are antecedents of emotion regulation strategies. Consistent with this view, we propose that emotion regulation strategies represent attempts by individual members to manage emotional dissonance and express their emotions following positive emotional display norms. Thus, members who perceive emotional dissonance engage in emotion regulation strategies to modify their emotions and return to a state of congruence (Diefendorff and Gosserand, 2003). When there is a difference between team members’ positive emotion and their positive emotional display norm, members feel psychologically anxious and experience stress (Festinger, 1957; Hochschild, 1983; Harmon-Jones and Mills, 1999). Furthermore, the greater the emotional dissonance, the more motivated the use of emotion regulation strategies to reduce dissonance (Grandey, 2000, 2003; Pugh et al., 2011). Therefore, we propose that the greater the incongruence between a team’s positive emotional display norm and a member’s positive emotion, the more team members will engage in emotion regulation strategies that can manage one’s positive emotion to reduce emotional dissonance.

Hypothesis 1a. The incongruence between the team’s positive emotional display norm and the team member’s positive emotion is positively related to deep acting.

Hypothesis 1b. The incongruence between the team’s positive emotional display norm and the team member’s positive emotion is positively related to surface acting.

### The moderation effect of self-regulatory focus

Self-regulation is the process of adjusting one’s behavior or self-concept to appropriate goals and standards (Brockner and Higgins, 2001). Higgins (1997) suggested that an individual’s regulation process is differentiated by needs, goals, and psychological situations. Promotion focus is directed at achieving ideal goals with development needs, and the presence of positive outcomes matters. In contrast, prevention focus is directed at achieving ought goals with safety needs, and the presence of negative outcomes matters. More specifically, when promotion-focused, individuals seek to align themselves with their ideal goals, whereas when prevention-focused, individuals make efforts to avoid misalignment with their ought goals. Depending on the nature of one’s own needs and goals, the self-regulatory process develops differently (Crowe and Higgins, 1997; Lanaj et al., 2012). Dewett and Denisi (2007) stated that the regulatory focus theory focuses on reducing the difference between the current state and the desired end state (Dewett and Denisi, 2007). If discrepancies between goals and self-concepts are detected, individuals attempt to reduce these differences. Thus, because self-regulation can explain an individual’s behavior toward a goal (Vriend et al., 2023), self-regulatory focus can be linked to an individual’s emotion-regulation behavior toward a goal. Based on this, we aim to examine how emotional regulation behaviors, as a strategy to reduce emotional dissonance between individual member’s positive emotions and the team’s positive emotional display norm, may differ depending on the regulatory focus.

Promotion-focused individuals tend to set ideal goals for positive outcomes based on their desire for growth and success (Koopmann et al., 2019). In addition, they tend to prioritize gain over loss and use approach-oriented strategies for the ideal goal, even if risk follows (Ahmadi et al., 2017). Therefore, promotion focus leads the team’s positive emotional display norms as an ideal goal (Brockner and Higgins, 2001; McMullen et al., 2009). Specifically, because the team’s positive emotional display norms are a social standard and part of the task to regulate an individual’s emotions (Grandey et al., 2012), promotion-focused individuals consider following the team’s positive emotional display norms the ideal goal (Bartel and Saavedra, 2000; Dewett and Denisi, 2007; Kim et al., 2022). Indeed, Van Dijk and Kluger (2011) reported that promotion focus tends to take risks for success and pursue change. Altogether, a high level of promotion focus will take the risk of changing one’s positive emotions and approach it to link one’s positive emotions with the ideal goal–team positive emotional display norms (Goldberg and Grandey, 2007; Diefendorff et al., 2011). Consequently, deep acting, which is expressing one’s positive emotions by changing them according to the team’s positive emotional display norms, is more likely from members with a high level of promotion focus. Thus, we came up with this hypothesis.

Hypothesis 2a. Promotion focus moderates the relationship between emotional dissonance and emotion regulation strategy. Specifically, the higher the level of promotion focus, the more the positive relationship between emotional dissonance and deep acting is strengthened.

Prevention-focused individuals tend to prioritize ought goals for the *status quo* (Bryant and Dunford, 2008). In addition, they often use a vigilance strategy to avoid negative outcomes such as failure based on security needs (Koopmann et al., 2019). Specifically, team members are under pressure in the context of conforming to team emotional display norms (Diefendorff et al., 2011; Becker and Cropanzano, 2015), and prevention-focused members consider team emotional display norms as ought goals with the duty of following them (McMullen et al., 2009; Higgins and Pinelli, 2020). In this regard, when team members perceive emotional dissonance, prevention-focused members can see the attempt to change positive emotions as risky choices and approach them differently from the team’s emotional display norms (Dahling and Johnson, 2013). Thus, the high level of prevention focus will try to prevent failure by adjusting one’s positive emotions to the team’s positive emotional display norms, with surface acting hiding one’s positive emotions and expressing them in line with the team’s positive emotional display norms. Based on this, we hypothesize as follows:

Hypothesis 2b. Prevention focus moderates the relationship between emotional dissonance and emotion regulation strategy. Specifically, the higher the level of prevention focus, the more the positive relationship between emotional dissonance and surface acting is strengthened.

### The effect of emotion regulation strategies on OCB

Research on emotion regulation indicates that observers can interpret members’ emotions expressed through deep acting and surface acting differently (Grandey, 2003; Grandey et al., 2005; Hülsheger and Schewe, 2011; Deng et al., 2020). Van Kleef (2009)’s Emotion as Social Information (EASI) theory describes the expressed emotion as containing information about the intention, purpose, and situation of the expressor. Based on this theory, the observer deduces the information in the emotion through inferential processing and forms a judgment and response. In addition, the observer’s judgment of these emotions affects the subsequent judgment of the expressor’s behavior (Van Kleef et al., 2012). Therefore, this study examines how observers interpret and evaluate the expressor’s OCB based on positive emotions expressed through emotion regulation strategies.

Organizational citizenship behaviors (OCB) are defined as behaviors that are indirectly related to tasks but voluntarily help and cooperate with other members (McNeely and Meglino, 1994). Rioux and Penner (2001) identified three motives for OCB: Prosocial values (PV), Organizational concerns (OC), and Impression management (IM). PV motives explain an employee’s motive to help and cooperate to have a positive relationship with others; OC motives explain an employee’s motive to help and belong to the organization; IM motives explain an employee’s motive to manage his or her own impression and avoid looking bad to others. Researchers have argued that OCB is not always a voluntary or intentional behavior for managing impressions (Bolino, 1999; Lee and Allen, 2002; Bolino et al., 2006). These researchers suggested that IM-motivated OCB is self-serving for instrumental gains; PV- and OC-motivated OCB is selfless (Rioux and Penner, 2001; Halbesleben et al., 2010). However, OCB appears in the form of helping members even if OCB is motivated by self-serving. Thus, the ratings of OCB may vary depending on how the observer interprets the OCB motivation (Van Kleef, 2014, 2016). Donia et al. (2016) reported that leader ratings varied depending on motivation for OCB. Given that observers have different interpretations of OCB according to the motive of a specific member, we expect to view the interpretation of a change in the motive of a specific member’s OCB, in particular, to examine how the observer’s rating varies according to the expressor’s positive emotion expressed through deep acting and surface acting.

Van Kleef (2014) shows that positive emotional expression can make an observer feel positive or have a positive impression of the expressor. However, not all positive emotions were positive. Observers can perceive differences in the authenticity of positive emotions expressed through emotion regulation strategies (Grandey, 2003; Grandey and Gabriel, 2015). Specifically, when interpreting positive emotions, the observer deduces the authenticity and interprets the expressor’s behaviors thereafter based on their responses to previous positive emotions (Forgas, 1995; Van Kleef, 2016). Deep acting involves changing one’s emotions into desired ones by consciously making efforts to explain their emotional experiences in accordance with the display rules. In contrast, surface acting involves faking and hiding one’s emotions (Grandey, 2000). For example, when a member’s level of positive emotion is low, if he or she tries to raise his or her level of positive emotion according to the display norm that requires a certain level of positive emotion expression, the member’s positive emotions will increase (Brotheridge and Lee, 2002; Grandey, 2003). Observers infer an expressor’s positive intention and purpose based on the expressor’s authentic positive emotional expression, which is then influenced when rating the expressor’s OCB (Ashforth and Humphrey, 1993; Grandey et al., 2005; Van Kleef et al., 2012). Therefore, the observer who perceived the positive emotional expression as authentic can judge the subsequent OCB as a selfless-motivated behavior (Knight and Eisenkraft, 2015; Donia et al., 2016; Van Kleef and Fischer, 2016; Parke and Seo, 2017). In contrast, if observers experience the inference process with inauthentic positive emotion through surface acting, they form a negative response to inauthentic emotions. Consequently, observers can judge subsequent OCB as a self-serving motivated behavior even if OCB is motivated by selfless motives. Hence, we propose the following hypotheses:

Hypothesis 3a. The expressor’s engagement in deep acting is positively related to the expressor’s selfless OCB perceived by the observer.

Hypothesis 3b. The expressor’s engagement in surface acting is negatively related to the expressor’s selfless OCB perceived by the observer.

## Materials and methods

### Participants and procedure

Previous EL research has focused on data on service jobs, but this research tried to increase the generalizability of our findings by collecting data on various jobs. For the objectives of this study, participants holding various jobs in South Korean organizations were invited. We collected our data at three time points and the survey consisted of two types: one for leaders and one for members. The leader survey was divided into three types, and the member survey was divided into two types. Both surveys included two common types. The first type measured the team’s positive emotional display norms, positive emotion, demographic information, and control variables at time 1. The second type consisted of emotion regulation strategies (deep acting and surface acting) and regulatory focus (promotion focus and prevention focus) at time 2. Finally, the third type of leader survey measured the leader’s evaluation of the member’s selfless OCB, which was conducted after all member surveys were collected. The first and second types of surveys were conducted 1°week apart, while the third type was conducted after all the second type surveys were collected. In total, 327 participants in 66 teams (93% response rate) took part in our survey. In the context of this study, a “team” is operationally defined as a group comprising three or more employees who engage in social interactions aimed at achieving interdependent goals (Kozlowski and Ilgen, 2006). Among the leaders, the average age was 43°years old (SD = 7.61), with 80% of them being men and 20% of them being women. The average age of the followers was 34 (SD = 8.27), with 58% of them being men. The majority of the teams (37%) worked in manufacturing organizations (finance: 10%, service: 19%, IT: 10%, others: 24%). According to Hox et al. (2010) and Peng et al. (2019), we chose to do grand mean centering.

### Measure

All items were measured on a 5-point Likert scale (1 = never to 5 = often).

#### Team positive emotional display norm

We assessed the team’s positive emotional display norms by Van Katwyk et al.’s (2000) Job-Related Affective Well-Being Scale (JAWS) at time 2. We viewed emotional display norms as team-level norms and used a referent-shift model (Klein et al., 2001; Diefendorff et al., 2011). A sample of items is “In our team, it is important for team members to experience and share their enthusiasm in the workplace.” (α = 0.87), and the items were aggregated to create a team-level score. In fact, before aggregating the display norms ratings, we examined the average r_*wg*_ (0.74) (Barcikowski, 1981; James et al., 1993).

#### Positive emotion

We assessed individual positive emotions with four items by JAWS (Van Katwyk et al., 2000) at time 2. These items asked the extent to which team members generally felt each positive emotion. The average Cronbach’s alpha was 0.88.

#### Emotion regulation strategies

We adopted 12 items from a scale by Diefendorff et al. (2005) at time 2. Participants rated deep acting with three items and surface acting with four items. A sample of the deep acting items is, “I make an effort to actually feel the emotions that I need to display toward others at work” (α = 0.78) and a sample of the surface acting items is, “I fake the emotions I show when dealing with team members” (α = 0.88).

#### Regulatory focus

We assessed promotion focus and prevention focus based on 10 items developed by Lockwood et al. (2002) at time 2. A sample of promotion focus items (four items) is, “I typically focus on the success I hope to achieve in the future” (α = 0.76). A sample of prevention focus items (six items) is “ I often worry that I will fail to accomplish my task goals” (α = 0.81).

#### Selfless motives of OCB

We assessed ratings of selfless OCB with four items (two items for each selfless OCB motivation: OC and PV) by Rioux and Penner’s (2001) Citizenship Motives Scale (CMS) at time 3. We viewed selfless OCB as a variable to examine how an observer interprets an expressor’s selfless OCB after the expressor’s emotion regulation strategy. To assess a member’s selfless OCB from the perspective of the observer, leaders were asked to think of each follower’s selfless OCB motivations and rate how certain motivations described the follower’s selfless OCB using a list of team members. A sample item is “The reason team member “A” helps during their daily work is that they feel it is important to help other team members in need.” (α = 0.73).

#### Control variables

We controlled for the individual’s age, rank (0 = managers; 1 = non-manager) at the individual level, and industry (0 = service; 1 = non-service) at the team level in the analyses. The distinction between service and non-service industries is based on whether employees interact with their customers at work (Grandey et al., 2015; Grandey and Melloy, 2017). Given that prior emotion regulation research suggested that team tenure and personality are related to deep acting and surface acting (Bono and Vey, 2005; Becker and Cropanzano, 2015; Grandey and Gabriel, 2015), we also controlled individual’s neuroticism and team tenure. As a personality factor, neuroticism was assessed with four items by McCrae and Costa (1987) (α = 0.81).

## Result

Table 1 shows means, standard deviations, and intercorrelations among the variables. Before testing our hypothesis, we conducted the confirmatory factor analysis (CFA). The CFA results indicated our seven major variables’ distinctiveness including team positive emotional display norms, individual positive emotion, deep acting, surface acting, promotion focus, prevention focus, and selfless OCB; χ2/*df* = 1.93, CFI = 0.92, TLI = 0.90, and RMSEA = 0.05 (Hypothesized model in Table 2).

**TABLE 1:** Means, standard deviations, and correlations.

Variable	Mean	SD	1	2	3	4	5	6	7	8	9	10	11
*Team level*													
1. Industry^a^	.84	.37											
2. Team positive emotional display norm	3.39	.52	-.19**										
*Individual level*													
3. Age	36.08	8.88											
4. Rank^b^	.62	.49			.56**								
5. Team tenure	3.54	62.87			.38**	.34**							
6. Neuroticism	2.59	.81			-.00	-.04	.02						
7. Positive emotion	3.35	.72			.11	.02	.04	-.22**					
8. Deep acting	2.72	.78			.08	.07	-.01	.03	.06				
9. Surface acting	2.66	.80			-.03	.00	-.03	.20**	-.17**	.43**			
10. Promotion focus	3.81	.55			-.10	-.03	.01	-.14**	.38**	.21**	-.01		
11. Prevention focus	2.91	.71			.02	-.02	.07	.31**	-.18**	.27**	.34**	-.12*	
12. OCB	2.72	.63			.06	.02	-.02	-.08	.18**	.52**	-.20**	.15*	.04

*N* = 66 (Team level); 327 (Individual level). SD = Standard Deviation.

^a^service industry = 0, non-service industry = 1;

^b^managers = 0, non-managers = 1.

*p < .05.

**p < .01.

**TABLE 2:** Confirmatory factor analysis.

Model	χ^2^/*df*	CFI	TLI	RMSEA
Hypothesized model^a^	1.93	.92	.90	.05
Alternative model 1^b^	2.86	.84	.80	.08
Alternative model 2^c^	2.88	.83	.79	.08

^a^Hypothesized model: Team positive emotional display norms (TPEDN), positive emotion (PE), deep acting (DA), surface acting (SA), promotion focus (PMF), prevention focus (PVF), and Organizational citizenship behaviors (OCB).

^b^Alternative model 1: TPEDN, PE, Emotion Regulation Strategy (DA + SA), PMF, PVF, and OCB.

^c^Alternative model 2: TPEDN, PE, DA, SA, Regulatory Focus (PMF + PVF), and OCB.

**FIGURE 2:**
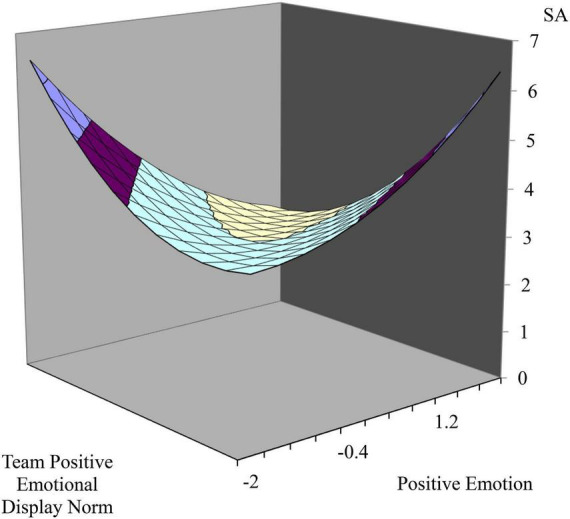
The incongruent effect on surface acting. The incongruent effect between team positive emotional display norm and positive emotion on surface acting.

Hypothesis 1 suggests an incongruence effect between team positive emotional display norms and individual positive emotion on emotion regulation strategies. Table 3 (H1a) and Table 4 (H1b) summarize multi-level polynomial hierarchical linear model (HLM) results. As shown in Table 3 (model 3), the interaction terms were not significant (γ_51_ = −0.23, *N.S.*). Thus, Hypothesis 1a was rejected. However, Table 4 shows that the second-order polynomial terms were significant (γ_51_ = −0.44, *p* < 0.05). Based on the results of the incongruence effect, we generated a three-dimensional response surface graph. Figure 2 indicated that when the team’s positive emotional display norms are higher than the individual’s positive emotion, and the individual’s positive emotion is higher than the team’s positive emotional display norms, surface acting was positively related to the incongruence as the response surface graph was curved downward along the incongruence line. Thus, hypothesis 1b was supported.

**TABLE 3:** The HLM results of deep acting.

Variable	Deep acting					
	**Model 1**	**Model 2**	**Model 3**	**Model 4**	**Model 5**	**Model 6**
Constant	2.69***	2.66***	2.59***	2.50***	2.49***	2.42***
Age	.01	.01	.01	.01	.01	.01
Rank^a^	.05	.05	.07	.08	.10	.12
Team tenure	-.00	-.00	-.00	-.00	-.00	-.00
Neuroticism	.05	.05	.05	.07	.07	.09
Industry^b^	-.04	-.01	-.00	.15	.09	.11
Individual Positive Emotion (PE)		.03	.06	-.04	-.03	-.02
Team Positive Emotional Display Norms (TPEDN)		.02	.00	-.01	-.01	.02
Promotion Focus (PMF)				.30**	.36**	.19
PE^2^			.12		.12	.18*
PE × TPEDN			-.23		-.36*	-.68**
TPEDN^2^			.12		.13	.34
PE × PMF					.07	.33*
TPEDN × PMF					.21	-.02
PE × TPEDN × PMF						-.00**
PE^2^ × PMF						.29*
TPEDN^2^ × PMF						.67*

N = 66 (Team-level); 327 (Individual-level). Model 1 - 3 is for examining hypothesis 1a; Model 4 - 6 is for examining hypothesis 2a.

^a^managers = 0, non-managers = 1;

^b^service industry = 0, non-service industry = 1.

*p < .05.

**p < .01.

***p < .001.

**TABLE 4:** The HLM results of surface acting.

Variable	Surface acting					
	**Model 1**	**Model 2**	**Model 3**	**Model 4**	**Model 5**	**Model 6**
Constant	2.95***	3.00***	2.90***	2.95***	2.89***	2.88***
Age	-.00	-.00	-.00	-.00	.00	-.00
Rank^a^	.07	.06	.09	.09	.08	.07
Team tenure	-.00	-.00	-.00	-.00	-.00	-.00
Neuroticism	.17*	.14*	.14*	.07	.07	.08
Industry^b^	-.39**	-.46**	-.46***	-.42**	-.40**	-.39**
Individual Positive Emotion (PE)		-.17*	-.14	-.11	-.12	-.09
Team Positive Emotional Display Norms (TPEDN)		-.13	-.14	-.10	-.08	-.08
Prevention Focus (PVF)				.33**	.30**	.29**
PE^2^			.17*		.08	.08
PE × TPEDN			-.44*		-.30	-.39*
TPEDN^2^			.27		.29	.35*
PE × PVF					-.09	-.09
TPEDN × PVF					.26	.30*
PE × TPEDN × PVF						.11
PE^2^ × PVF						.03
TPEDN^2^ × PVF						-.04

N = 66 (Team level); 327 (Individual level). Models 1 - 3 are for examining hypothesis 1b, and Models 4 - 6 are for examining hypothesis 2b.

^a^managers = 0, non-managers = 1;

^b^service industry = 0, non-service industry = 1.

*p < .05.

**p < .01.

***p < .001.

**FIGURE 3:**
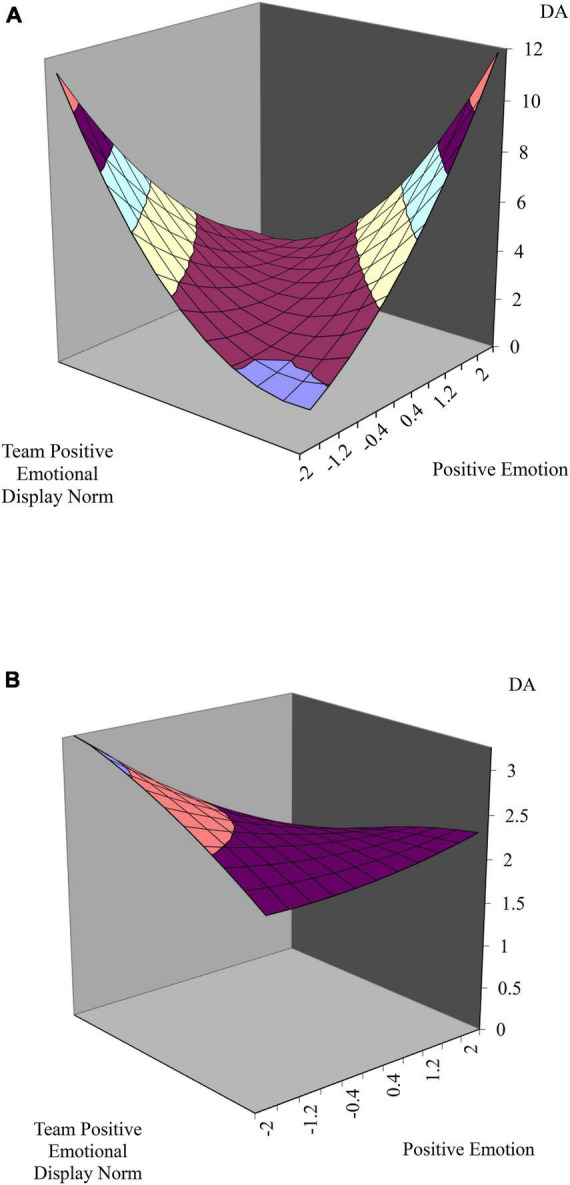
**(A)** The relationship between emotional dissonance and deep acting with moderation effect of promotion focus. The incongruent effect between team positive emotional display norm and individual positive emotion on deep acting when the level of promotion focus is high. **(B)** The relationship between emotional dissonance and deep acting with moderation effect of promotion focus. The incongruent effect between team positive emotional display norm and individual positive emotion on deep acting when the level of promotion focus is low.

Hypothesis 2 suggests the moderation effect of regulatory focus on the relationship between emotional dissonance and emotion regulation strategies. To test moderated multi-level polynomial regression, we incorporated the moderator variable into a quadratic regression equation (Edwards and Parry, 1993; Edwards, 2002). According to Edwards (2002), the compound coefficients on the five terms (**X**, **Y**, **X***Y*, **X^2^** and **Y^2^**) of the equation including the moderator variable can be used to test weighted linear combinations. First, hypothesis 2a predicted that the relationship between emotional dissonance and deep acting is moderated by promotion focus. Table 3 in model 6 indicated that two second-order interaction terms were significant (γ_72_ = 0.67, *p* < 0.05; γ_90_ = 0.29, *p* < 0.05). Given these results, we generated two response surface graphs by substituting ± 1 standard deviation of promotion focus (± 0.55) for V in the regression equation (Edwards, 2002). Figure 3A shows a deeper U-shaped curve along the incongruence line (*b*_1_ – *b*_2_ = 0.15, *p* = *N.S*; *b*_3_ – *b*_4_ + *b*_5_ = 2.29, *p* < 0.00) than Figure 3B (*b*_1_ – *b*_2_ = −0.23, *p* < 0.1; *b*_3_ – *b*_4_ + *b*_5_ = 0.12, *p* = *N.S*.). In this regard, promotion focus moderates the relationship between emotional dissonance and deep acting, supporting Hypothesis 2a.

**FIGURE 4:**
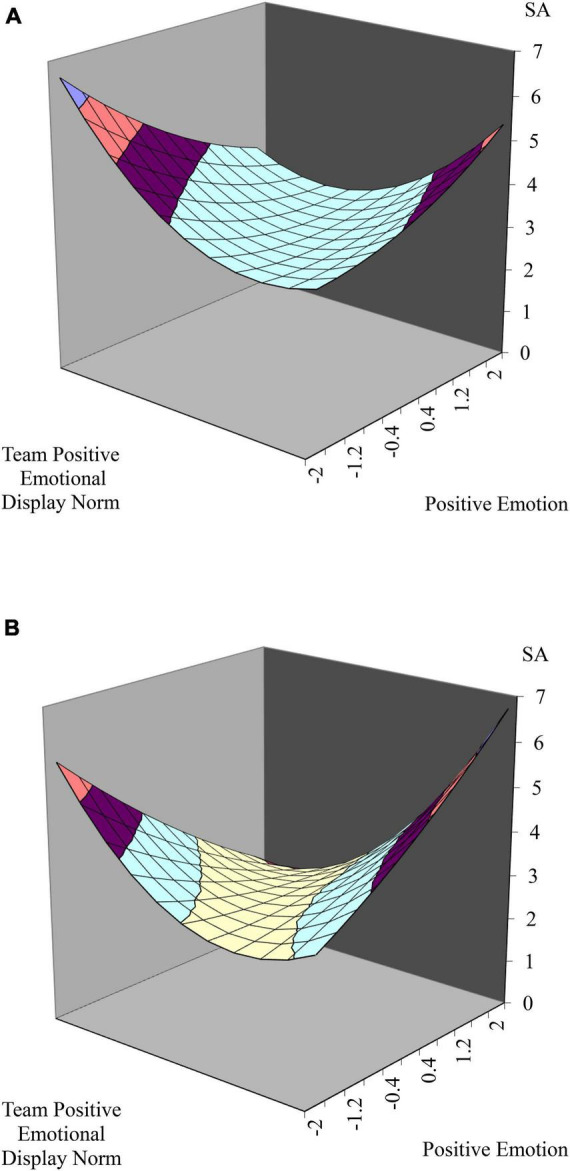
**(A)** The relationship between emotional dissonance and surface acting with moderation effect of prevention focus. The incongruent effect between team positive emotional display norm and individual positive emotion on surface acting when the level of prevention focus is high. **(B)** The relationship between emotional dissonance and surface acting with moderation effect of prevention focus. The incongruent effect between team positive emotional display norm and individual positive emotion on surface acting when the level of prevention focus is low.

Hypothesis 2b predicted that prevention focus moderates the relationship between emotional dissonance and surface acting. The interaction term was found to be significant (γ_71_ = 0.30, *p* < 0.05) in Table 4 (model 6). Similar to hypothesis 2a, two response surface graphs were generated by substituting ± 1 standard deviation of prevention focus (± 0.71) in the regression equation. Figures 4A, B show that the surface along the PE = - TPEDN line curved downward. Specifically, Figure 4A indicates that when team positive emotional display norm is higher than individual positive emotion, surface acting is rapidly increasing than in Figure 4B. Moreover, we find the significant curvatures of both Figure 4A (*b*_1_ – *b*_2_ = 0.73, *p* < 0.00; *b*_3_ – *b*_4_ + *b*_5_ = 0.73, *p* < 0.00) and Figure 4B (*b*_1_ – *b*_2_ = 0.26, *p* < 0.05; *b*_3_ – *b*_4_ + *b*_5_ = 0.90, *p* < 0.00) along the incongruence line, supporting Hypothesis 2b.

To test Hypothesis 3, we generated block variables using the quadratic equations used to examine Hypothesis 2, which consists of two variables for deep acting and surface acting through the five terms (**X**, **Y**, **X***Y*, **X^2^** and **Y^2^**) in the regression equations, respectively (Edwards, 2001; Edwards and Cable, 2009). Because the block variable is produced from the estimated coefficient, the deviation using the block variable in an equation is the same in describing the original second-order equation (Lambert et al., 2012; Zhang et al., 2012; Matta et al., 2015; Kim et al., 2022). Hypothesis 3a predicted a positive relationship between deep acting and selfless OCB. As a result, selfless OCB was not significantly related to the relations (*b* = 0.01, *N.S.*), thereby rejecting Hypothesis 3a. Similarly, Hypothesis 3b predicted a negative relationship between surface acting and selfless OCB. As a result of conducting regression analysis after constructing a block variable, it was found that the relationship between surface acting and selfless OCB had a negatively significant relationship (*b* = −0.75, *p* < 0.05). Hypothesis 3b was, therefore, supported.

## Discussion

### Theoretical implications

First, the team positive emotional display norm in this study is the team-level display rule shared among team members. Previous studies on display rules have mostly focused on how members perceive and conform to the display rules from the top down, such as organizational requirements, at the individual level (Hochschild, 1983; Goldberg and Grandey, 2007; Diefendorff et al., 2019). Studies examining the influence of display rule at the team level are rare. However, this study empirically showed that the display norms formed by top-down and bottom-up can be shared among team members. Furthermore, it confirmed that the more the team’s positive emotional display norms do not fit the individual positive emotions, the more it affects emotion regulation strategies, which can also affect the observer’s perspective on selfless OCB evaluation. Thus, this study contributes to the understanding of display rules at the team level, examining the direct and indirect effects of team positive emotional display norms on the emotion regulation process of team members.

Second, based on multi-level polynomial regression analysis and three-dimensional graphs, we examined the effect in the form of emotional dissonance that occurs when positive emotional display norms at the team level and individual positive emotions are incongruent. Previous studies mainly argued that emotional dissonance was a difference value perceived by individuals through a direct measurement method (Coté, 2005; Gosserand and Diefendorff, 2005). However, in this study, using the difference value of the indirect measurement method, individual members’ positive emotions and the team’s positive emotional display norms were measured, respectively. Results of the analysis prove that emotional dissonance can be formed not only when the team’s positive emotional display norm is higher than the member’s positive emotion but also when the individual member’s positive emotion is higher than the team’s positive emotional display norm. Accordingly, this study expanded the scope of research on emotional dissonance in that it found that each person reacts differently to the form of emotional dissonance.

Third, this study examined the relationship between emotional dissonance and deep acting, as well as the relationship between deep acting and evaluations of selfless OCB. In previous emotion regulation research, the results regarding the relationship between emotional dissonance and deep acting, as well as the effect of deep acting, have not been consistent (Bono and Vey, 2005; Grandey and Gabriel, 2015; Huppertz et al., 2020). The results of this study revealed that deep acting can be considered the preferred emotion regulation strategy depending on individual tendency (promotion focus) (H2a) even if it does not have a significant positive relationship with emotional dissonance (H1a). Moreover, the leader’s interpretation of the team member’s genuine positive emotion is not positively related to the rating of the member’s selfless OCB even when the member may genuinely express positive emotion through deep acting (H3a). Manokara et al. (2023) found that positive emotions are not shown in the same way and the display rules differ between positive emotions. In other words, the outcomes can vary depending on which specific positive emotion is expressed. The present research thus contributes to the existing literature on emotion management and display norms by examining the antecedents and effects of deep acting, thereby shedding light on the process of deep acting (Zapf et al., 2021; Nesher Shoshan and Venz, 2022).

Lastly, this study focuses on the effect of positive emotion through emotion regulation strategies on selfless OCB from the observer’s perspective. The study’s findings indicate that the expressor’s positive emotion through deep acting is not positively related to the observer’s evaluation of selfless OCB. In contrast, when the expressor engages in surface acting, it is negatively associated with the observer’s rating of selfless OCB. This suggests that observers may not interpret the expressor’s positive emotion positively through either emotion regulation strategy, but positive emotion through surface acting strategy may negatively affect the rating of selfless OCB. Thus, it is important to understand that observers base their interpretation of the expressor’s future behavior on their own responses to the expressor’s emotion.

### Practical implications

First, the study explored emotional labor within teams based on interactions among team members. Although there are previous studies on emotional labor experiences in interactions with external customers (Ashforth and Humphrey, 1993; Côté and Morgan, 2002; Diefendorff et al., 2019), studies on emotional labor experiences in interactions among team members are insufficient (Diefendorff et al., 2020). Accordingly, we demonstrate the need for organizations and managers to manage team positive emotional display norms for effective emotional management of team members since members who constantly interact to achieve a common goal manage their emotions based on the team’s positive emotional display norms. Furthermore, organizations may need to provide training as a necessity for the team’s positive emotion experience standards such that team members can effectively manage their emotions by setting them as the standard for expressing their own emotions.

Second, organizations may need to differentiate members’ emotion management training according to individual tendencies such as self-regulatory focus. The higher the level of promotion focus, the more individuals regulate their emotions through deep acting to approach the goal of display norms. Deep acting can cause stress in the process of regulating positive emotions to fit one’s ideal goal and the team’s positive emotional display norms. On the other hand, the higher the prevention focus, the more individuals hide their emotions and express false positive emotions to prevent emotion regulation failure based on surface acting. Accordingly, organizations need to provide a program for managing the stress that occurs after emotional regulation for individuals with a high promotion regulatory focus. On the other hand, for individuals with a high prevention focus, organizations need to de-emphasize disadvantages such as penalties for failure in the process of setting and managing goals. For example, a program that can positively view failure and encourage challenges such that they can find a solution through failure rather than being afraid of failure may be helpful.

### Limitations and future research directions

First, by examining the shared display norms at the team level, this study suggests that team members performing interdependent tasks share and follow the norms through interaction. However, the boundaries for teams are becoming blurred as the number of project teams and virtual teams increases (Sarker et al., 2011). In this study, the traditional team that continuously interacted in the same space was targeted, but future research might conduct research on new types of teams. Glikson and Erez (2013) suggested the emergence of emotional display norms in multicultural virtual teams with global identity. In this regard, future research on display norms might examine various emotional display norms that appear in new types of teams as well as traditional types of teams.

Second, Hypothesis 1a predicted a positive relationship between emotional dissonance and deep acting but it was rejected. The factors that may affect the effect of emotional dissonance on deep acting are likely to be related to other dispositional factors such as regulatory focus. In this study, regulatory focus was measured as an individual dispositional factor. Thus, future research could explore the phenomenon of preference for specific regulatory strategies using various dispositional variables. Furthermore, future research may need to consider the level of sharing or the degree to which members perceive it as role behavior even when team positive emotional display norms are shared among team members.

Finally, the purpose of this study was to examine the effect of emotional dissonance caused by the difference between the team’s positive emotional display norms and the member’s positive emotions. However, managing the emotions of team members is not limited to positive emotions, and negative emotions need to be managed (To et al., 2021). In addition, unlike when the observer interprets the positive emotion of a specific member when the negative emotion is interpreted, the effect on the evaluation of the subsequent behavior may be different. Thus, this study suggests looking at the emotional labor process of team members not only with positive emotions but also with negative emotions. Based on this, it is expected that it will be expanded to research on the team emotional display norms that include both positive and negative emotions as standards for emotion regulation of team members.

## Data availability statement

The raw data supporting the conclusions of this article will be made available by the authors, without undue reservation.

## Ethics statement

Ethical approval was not required for the studies involving humans because this study was conducted after providing sufficient information to the participants and obtaining their consent regarding the survey in accordance with the local legislation and institutional requirements. The studies were conducted in accordance with the local legislation and institutional requirements. The participants provided their written informed consent to participate in this study.

## Author Contributions

DH: Methodology, Resources, Writing – original draft, Writing – review and editing. MK: Conceptualization, Supervision, Writing – review and editing, Methodology.
